# Evaluation of landslide susceptibility based on SMOTE-Tomek sampling and machine learning algorithm

**DOI:** 10.1371/journal.pone.0323487

**Published:** 2025-05-21

**Authors:** Ming-zhou Lv, Kun-lun Li, Jia-zeng Cai, Jun Mao, Jia-jun Gao, Hui Xu

**Affiliations:** 1 School of Civil Engineering and Architecture, Zhejiang Sci-Tech University, Hangzhou, China; 2 Zhenan Comprehensive Engineering Surveying and Mapping Institute, Lishui, China; 3 Hangzhou Guowei Construction Engineering Corporation Limited, Hangzhou, China; China Construction Fourth Engineering Division Corp. Ltd, CHINA

## Abstract

Landslides are frequent and hazardous geological disasters, posing significant risks to human safety and infrastructure. Accurate assessments of landslide susceptibility are crucial for risk management and mitigation. However, geological surveys of landslide areas are typically conducted at the township level, have lowsample sizes, and rely on experience. This study proposes a framework for assessing landslide susceptibility in Taiping Township, Zhejiang Province, China, using data balancing, machine learning, and data from 1,325 slope units with nine slope characteristics. The dataset was balanced using the Synthetic Minority Oversampling Technique and the Tomek link undersampling method (SMOTE-Tomek). A comparative analysis of six machine learning models was performed, and the SHapley Additive exPlanation (SHAP) method was used to assess the influencing factors. The results indicate that the machine learning algorithms provide high accuracy, and the random forest (RF) algorithm achieves the optimum model accuracy (0.791, F1 = 0.723). The very low, low, medium, and high sensitivity zones account for 92.27%, 5.12%, 1.78%, and 0.83% of the area, respectively. The height of cut slopes has the most significant impact on landslide sensitivity, whereas the altitude has a minor impact. The proposed model accurately assesses landslide susceptibility at the township scale, providing valuable insights for risk management and mitigation.

## Introduction

Landslide hazards are prevalent globally, with high occurrence rates in numerous regions [1]. They present significant threats to human life and property, causing considerable socioeconomic losses every year. Therefore, analyzing the susceptibility of landslides is crucial for environmental protection, urban planning, resource optimization, and safeguarding human life and property. Extensive geological surveys of historical landslide areas have been conducted to analyze and predict landslide susceptibility and determine influencing factors, such as topographic, geomorphological, hydrological, and geological conditions. The results of landslide susceptibility analyses are used for landslide risk assessment, regional disaster mitigation and prevention, and landslide management. The scale of geological surveys has shifted from large to medium and small scales to achieve higher precision and prevent geological disasters. An increasing number of landslide studies have focused on the township level. This study performs landslide susceptibility at this level.

Existing studies have provided substantial insights into landslide susceptibility analysis [2,3]. Early research primarily focused on correlation analyses of influencing factors. With the advancement of computer technology and statistical theory, an increasing number of models have been utilized for the quantitative analysis of landslide susceptibility [4]. Hierarchical analysis has been widely used to map slope susceptibility [5]. Trisnawati et al. used a weighting method and the analytic hierarchy process to evaluate landslide susceptibility in Pringapus, East Ungaran, and evaluated the method’s accuracy [6]. Sharma and Mahajan used frequency ratios to map landslide susceptibility in the Indian Himalayan watershed and compared the results with those of the analytic hierarchy process [7]. Researchers have invested considerable effort and have accumulated valuable experience. However, a lack of quantitative results exists to substantiate their findings. Due to advances in computer technology, machine learning models have received increased attention for assessing landslides, including logistic regression (LR), support vector machines (SVMs), random forest (RF), and artificial neural networks (ANNs). These models are increasingly used to improve the accuracy and reliability of analyzing influencing factors. Fang and Wang utilized a deep neural network to predict landslide susceptibility in the Three Gorges Reservoir area [8]. Hussain et al. analyzed the correlation between landslides and geo-environmental factors using three tree-based classifiers: extreme gradient boosting (XGBoost), RF, and k-nearest neighbors (KNN). Their findings demonstrated the potential of these algorithms for urban and infrastructure planning [9]. Sun et al. advanced this field by employing Bayesian optimization with light gradient boosting machine (GBM) models, significantly improving the accuracy of the test and training sets compared to traditional models [10]. Similarly, Chen et al. applied gradient boosting decision trees (GBDTs), RF, and an information value model (IVM) for landslide susceptibility mapping in the Three Gorges Reservoir area. The GBDT outperformed the other methods [11]. These studies highlight the effectiveness of machine learning techniques in landslide susceptibility assessments. However, these models typically require extensive datasets, significantly increasing the data collection and survey workload. Although considerable attention has been devoted to optimizing model performance, the importance of susceptibility assessment has often been overlooked.

Due to the implementation of precise geological disaster prevention policies, landslide assessments have increasingly focused on relatively small areas, such as counties and villages. Faming Huang et al. studied Xunwu County in Ganzhou City, Jiangxi Province. When the random missing of landslide logging data exceeds 50%, it may affect the predictive ability of machine learning models, especially in areas with missing landslide samples [12]. As a result, a common challenge in small-scale landslide sensitivity assessments is the limited amount of historical landslide data available for model training, potentially reducing prediction accuracy [13]. In many cases, the number of observed landslide events is substantially smaller than that of non-landslide samples, significantly affecting the accuracy and reliability of susceptibility assessments. Scholars have proposed solutions to address sample imbalance. For instance, Xu et al. proposed selecting non-landslide samples based on their distance to landslides to achieve balanced datasets; however, the randomness of this method can affect the accuracy of susceptibility modeling [14]. Li et al. employed undersampling techniques to cluster pixels of non-geological disasters and selected central pixels corresponding to the number of disaster points. However, this approach was influenced by regional differences [15]. Faming Huang et al. studied the optimization method under the condition of sample imbalance in a certain area of Jiujiang, Jiangxi Province, China, and proposed a semi supervised imbalance theory, which greatly improved the prediction accuracy of landslide susceptibility. However, relying solely on ROC to determine the prediction accuracy is not reliable enough [16]. Heckmann et al. found that increasing sample sizes improved model performance [17]. The scarcity of landslide samples and the abundance of non-landslide samples often lead to model bias and overfitting [18]. In summary, the sample balance affects landslide susceptibility assessments and model accuracy. Researchers have focused on two main strategies to address this issue. The first approach involves sample optimization. Castro and Braga introduced a cost-sensitive algorithm (CSMLP) to balance decision boundaries in the sample’s feature space [19]. Galar et al. noted that combining random undersampling with bagging or boosting yielded high performance for imbalanced datasets [20]. The second optimizes the dataset by undersampling and oversampling to balance the data distribution. However, undersampling can lead to information loss, negatively affecting classifier performance [21]. Oversampling may cause data redundancy and contribute to overfitting [22]. The dataset optimization method depends on the research area and sampling method. For instance, Elhassan et al. utilized Tomek and random undersampling to deal with unbalanced data; however, this method resulted in information loss in some categories [23]. Chawla et al. proposed the Synthetic Minority Oversampling Technique (SMOTE) to synthesize and balance the data and reduce overfitting [24]. Although this method is sensitive to noise, it increases the number of samples in the minority class by utilizing existing samples and augmented data. Seiffert et al. [25] and Meng and Li [26] demonstrated that mixed sampling resulted in higher classification accuracy than single sampling methods. Recent evidence from Penang, Malaysia, indicates that the SMOTE-edited nearest neighbor (ENN) resampling algorithm combined with RF accurately predicted landslide sensitivity despite a scarcity of landslide samples [27].

In summary, small-scale geological surveys and landslide susceptibility assessments are major trends due to their low cost, high applicability, and adaptability. Data sampling techniques are commonly employed in cases of small sample sizes and class imbalances in small regions. Oversampling, undersampling, and mixed sampling have been used [28]. Undersampling aims to balance the dataset by reducing the number of majority-class samples to approach the number of minority-class samples. Conversely, oversampling seeks to balance the dataset by increasing the number of minority class samples to approximate the number of majority class samples. However, undersampling may cause a loss of potentially valuable information, whereas oversampling can increase the risk of model overfitting. Both have drawbacks [29]. Building on these findings, our study employs the SMOTE-Tomek sampling method, which combines SMOTE oversampling with Tomek link undersampling. The strength of this approach lies in its ability to not only generate synthetic samples for the minority class but also to remove noisy and redundant samples from the majority class. This enhances the model’s ability to capture more meaningful feature information. We then evaluate landslide susceptibility in Taiping Township, Liandu District, Zhejiang Province, China, using six machine learning models. In order to improve the interpretability of the results, We employ SHapley Additive exPlanation (SHAP) to analyze the contribution and importance of features in the susceptibility assessment results. This research fills a gap in landslide susceptibility assessments at large scales and ensures the accurate prediction of regional geological hazards.

## Study area and data acquisition

### Study area

**Fig 1:**
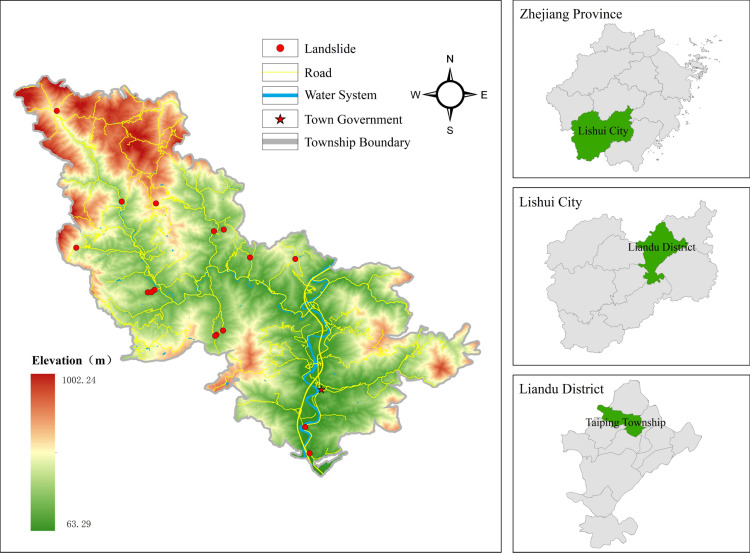
Distribution of historical landslides in the study area. (Republished from the data provided by Zhejiang Zhenan Comprehensive Engineering Surveying Institute under a CC BY license).

The study area is Taiping Town in the Liandu District of Lishui City, Zhejiang Province, China (119.91° E and 28.45° degrees N) (Fig 1). This region has a hilly topography and a subtropical monsoon climate characterized by abundant rainfall influenced by typhoons from the Pacific Rim. These conditions make it particularly susceptible to landslide disasters. Extensive rock weathering and the prevalence of geological faults contribute to this vulnerability.From year 2016–2020, Zhejiang Province experienced 1,096 landslide incidents, resulting in 48 fatalities and direct economic losses of 229 million yuan. In Taiping Town, 795 potential landslide sites exist, posing a threat to approximately 22,000 residents and a potential economic loss of 1.22 billion yuan. Additionally, human activities, including resource development and construction projects in Taiping Township, can exacerbate the natural factors, increasing the frequency of landslide disasters.

### Data acquisition

The landslide data were sourced from the local government and field investigations. According to the historical disaster records and remote sensing interpretations, 16 landslide incidents occurred in Taiping Township by the end of June 2023. Most incidents were observed on low slopes and in the weathered layers of steep slopes. Landslides with volumes exceeding 10,000 m^3^ accounted for 17.65%, and those with volumes below 7,000 m^3^ comprised 82.35%. The majority were small landslides attributed to engineering activities. The Taiping Township government provided supplementary data, including geological maps, rainfall records, road vectors, and geological information.

**Fig 2:**
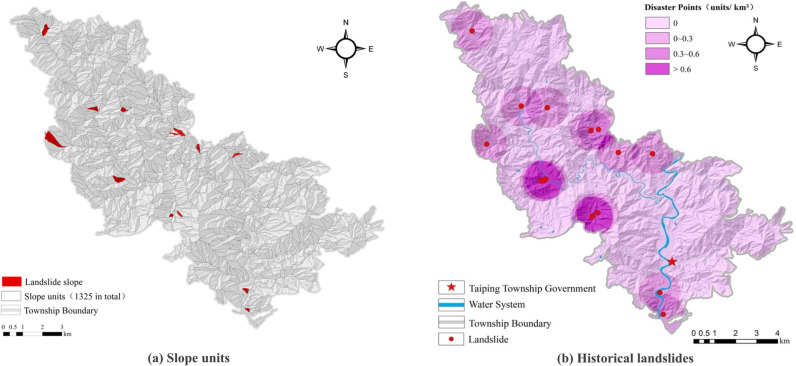
Slope unit division and historical landslide distribution. (Republished from the data provided by Zhejiang Zhenan Comprehensive Engineering Surveying Institute under a CC BY license).

Landslide susceptibility assessments generally employ slope or raster units for predictions. The evaluation unit can significantly affect the outcome. Therefore, selecting the most appropriate evaluation unit for landslide susceptibility analysis is critical. The small area of Taiping Township necessitated the use of slope units. This approach considers topographic conditions, and the unit is closely linked to geo-environmental factors. We used a digital elevation model (DEM) to identify 1,325 slope units (Fig 2(a)). We delineated 16 historical landslide sites (Fig 2(b)). This dataset provided the foundation for conducting the landslide susceptibility evaluations in the study area, with slope units as the primary prediction unit. The susceptibility index was obtained from the sample data. Machine learning models were used for predictions, and ArcGIS software was used for spatial analysis. Natural breakpoints were utilized to classify the landslide susceptibility zones.

An extensive review of the literature indicates a complex nonlinear relationship between landslide occurrence and geological factors, including topography, geological structure, rock and soil properties, and overburden thickness. Landslide disasters are often triggered by external factors, such as rainfall and slope cutting for housing and road construction. Based on the characteristics of Taiping Township and data availability, we initially identified nine independent influencing factors (Table 1): lithology and geotechnical structure, slope type, overburden thickness, slope gradient, primdifference, slope aspect, slope shape, distance to faults, and the height of cut slopes. The lithology and geotechnical structure, slope type, and overburden thickness were derived from engineering surveys and field investigations. A 30-meter resolution DEM was utilized to determine altitude differences, slope gradients, aspects, slope shapes, and distances to faults using ArcGIS software. The height of cut slopes was obtained from field surveys.

**Table 1:** Landslide evaluation factors and value range.

Evaluation factor	Data type	Definition and value range
X_1_ lithology and geotechnical structure	Vector	Classified according to structural changes
		Degree of fragmentation
		Structural type of the rock body
X_2_ slope type	Raster	Near-horizontal stratified slope
		Oblique slope
		Reverse slope
		Dip slope
X_3_ overburden thickness (m)	Raster	<1
		1 ∼ 3
		3 ∼ 6
		6 ∼ 12
		12 ∼ 25
		>25
X_4_ distance to faults (m)	Vector	<50
		50 ∼ 100
		100 ∼ 300
		300 ∼ 500
		>500
X_5_ slope(°)	Raster	<15
		15 ∼ 25
		25 ∼ 35
		35 ∼ 45
		>45
X_6_ altitude difference (m)	Raster	<20
		20 ∼ 50
		50 ∼ 100
		100 ∼ 300
		>300
X_7_ slope aspect (°)	Raster	North (337.5 ∼ 22.5)
		North East (22.5 ∼ 67.5)
		East (67.5 ∼ 112.5)
		South East (112.5 ∼ 157.5)
		South(157.5 ∼ 202.5)
		South West (202.5 ∼ 247.5)
		West (247.5–292.5)
		North West (292.5–337.5)
X_8_ slope shape	Raster	Conx slope
		Concave slope
		Straight slope
X_9_ height of cut slopes (m)	Raster	<3
		3 ∼ 6
		6 ∼ 12
		12 ∼ 25
		>25

### Data analysis

#### Correlation analysis.

**Fig 3:**
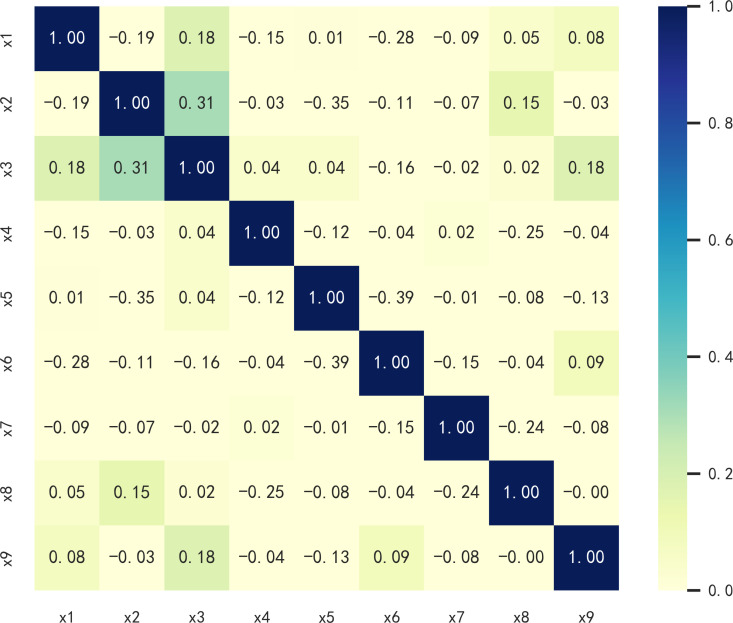
Correlation results of landslide evaluation factors.

Spearman correlation analysis was conducted to evaluate the correlation among the nine influencing factors [30,31]. It is a non-parametric rank correlation method that assesses the strength and direction of association between two variables. Spearman’s rank correlation coefficient $\rho_{s}$ is defined as:


$$\rho_{s}=1-\frac{6\sum d_{i}^{2}}{n(n^{2}-1)}$$ (1)

where *d*_*i*_ is the difference between the ranks of the values in the two datasets (for the *i*-th pair), and *n* is the number of data pairs. The results were used to select factors for assessing landslide susceptibility. The correlation analysis results of the nine landslide evaluation factors are presented in Fig 3. The absolute value of the correlation coefficient indicates the correlation strength. Values closer to 1 indicate a higher correlation, and those closer to 0 suggest a lower correlation. The absolute values of the correlation coefficient between the nine factors are relatively low; most are below 0.1, indicating a low correlation. Consequently, all nine landslide evaluation factors can be utilized for the subsequent susceptibility assessment. The nine evaluation factors were classified using established standards.

#### SMOTE-Tomek sampling.

SMOTE oversampling and Tomek Link undersampling were employed to mitigate sample imbalance in landslide susceptibility assessments. This combination improves the accuracy of model training and more accurately reflects the sample distribution of different classes. In addition, more minority samples are created while removing overlapping samples between categories, providing a more balanced dataset for model training [32]. The SMOTE algorithm was used to increase the number of minority class samples using data augmentation. The Tomek method was utilized to remove majority class samples close to the minority class samples to reduce noise in the synthetic data. The steps of the SMOTE algorithm are as follows:

(1) It is assumed that the set of minority class samples in the sample dataset is *S*_*min*_. For each sample *x*_*i*_ in *S*_*min*_, calculate its Euclidean distance to all other data samples in *S*_*min*_ and the KNN;
(2) Determine the appropriate sampling multiplier N according to the imbalance ratio of the categories;
(3) For each minority class sample *x*_*i*_, randomly select multiple samples *x*_*n*_ from its KNN;
(4) For each randomly selected *x*_*n*_, construct a new minority class data sample *x*_*new*_ as follows.

$$x_{new}=x_{i}+rand(0,\;1)*|x_{n}-x_{i}|$$ (2)

**Fig 4:**
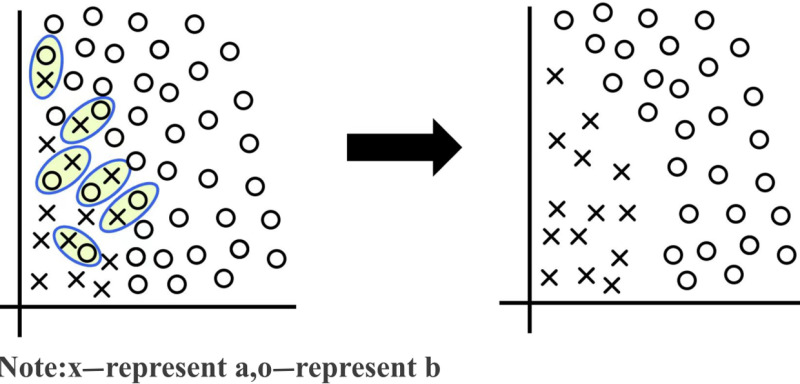
Schematic diagram of the Tomek link.

Subsequently, the dataset was enhanced using the Tomek Link method, which removes all samples in the majority class in the Tomek Link pairs. A set of nearest-neighbor samples from different classes is defined as a Tomek Link (Fig 4). These samples may represent boundaries between classes and may contain noise. The objective of this step is to define decision boundaries and eliminate noise by identifying an removing samples from majority and minority classes that may cause the classifier to degrade while reducing the risk of overfitting and enhancing the robustness of the dataset [33]. The initial dataset contained 1,325 samples, including 16 landslide samples and 1,309 non-landslide samples. The synthetic landslide samples were generated using the SMOTE method, increasing the landslide samples to 1,309. Subsequently, Tomek Link undersampling was performed to ensure that the number of non-landslide and landslide samples matched, resulting in 259 landslide samples, accounting for 16.5% of the dataset. Landslide sample ratios of 10% to 20% typically yield good performance in machine learning models [34,35]. The final number of data samples is presented in Table 2.

**Table 2:** Number of samples after dataset optimization.

Sampling method	Non-landslide samples	Landslide samples	Number of modeling samples
**Initial data**	1309	16	1325
**SMOTE**	1309	1309	2618
**SMOTE-Tomek**	1309	259	1568

#### Kruskal-Wallis test.

**Fig 5:**
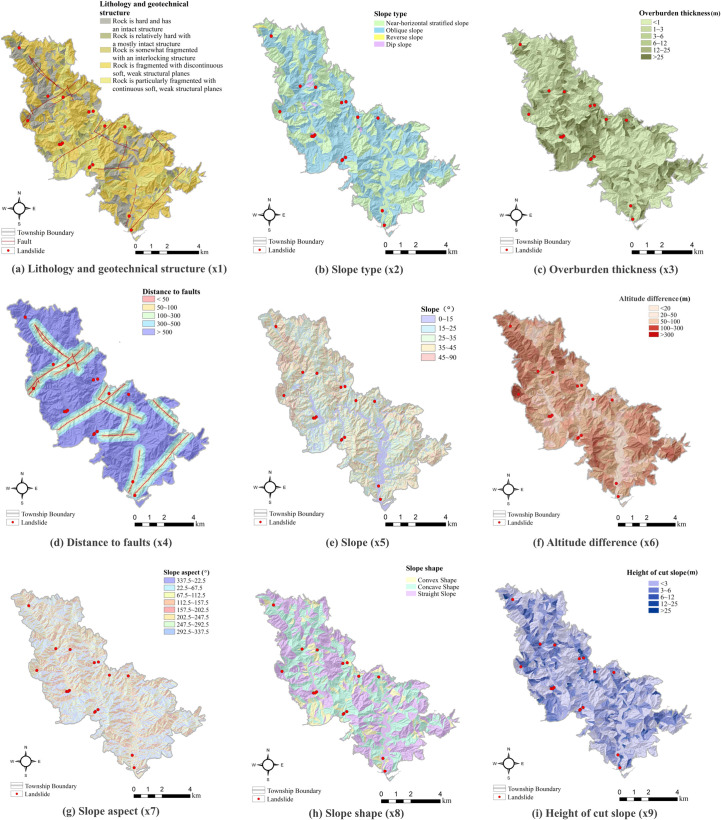
Distributions of landslide evaluation factors. (Republished from the data provided by Zhejiang Zhenan Comprehensive Engineering Surveying Institute under a CC BY license).

After applying SMOTE-Tomek sampling, a Kruskal-Wallis test was conducted on the distribution of landslide evaluation factors. The results are listed in Table 3. The KW test is a non-parametric statistical method used to compare more than two independent groups, making it ideal for datasets that do not follow a normal distribution. Its advantage lies in its ability to assess whether there are statistically significant differences between the groups without making assumptions about the data’s underlying distribution. In this study, it was used to assess whether mixed sampling caused statistically significant changes in parameter values between landslide and non-landslide samples. The results indicated significant differences in several features between the groups, demonstrating the effectiveness of the mixed sampling approach in balancing the dataset. All p-values were below 0.05, indicating that the mixed sampling technique was able to detect significant differences between landslide and non-landslide samples. The distributions of the nine evaluation factors in are shown in Fig 5.

**Table 3:** Results of Kruskal-Wallis test.

Evaluation factor	Statistic	p-value
**x1**	921.628	0.000*
**x2**	273.429	0.000*
**x3**	1304.627	0.000*
**x4**	3.902	0.048*
**x5**	476.708	0.000*
**x6**	337.962	0.000*
**x7**	91.411	0.000*
**x8**	19.761	0.000*
**x9**	803.461	0.000*

## Landslide susceptibility assessment method

**Fig 6:**
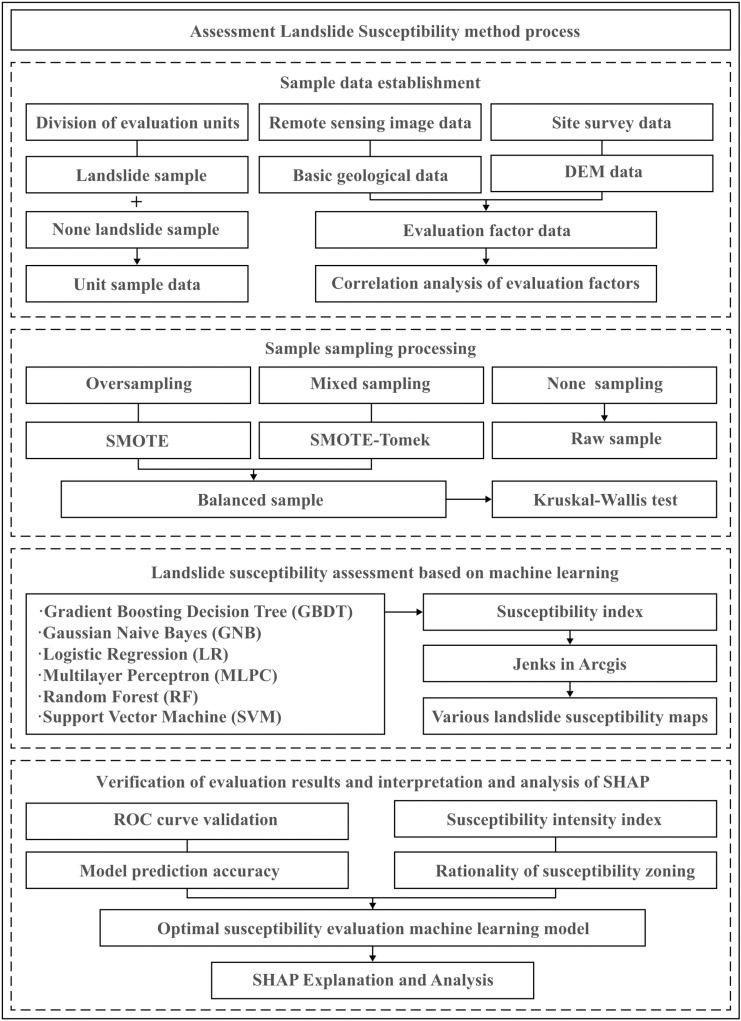
Workflow of landslide susceptibility assessment.

This section describes the methodology and modeling framework for slope susceptibility analysis, as well as the evaluation methodology. Six machine learning algorithms were employed: RF, GBDT, the Gaussian naive Bayes (GNB) algorithm, LR, multilayer perceptron (MLPC), and SVM. The receiver operating characteristic curve (ROC), accuracy, and F1 score were utilized to evaluate the model’s performance, and the rationality of the results was validated based on the susceptibility intensity index. The SHAP model was used to assess the contributions of the evaluation factors, providing insights into the interpretability of the machine learning approaches. The workflow of this study is illustrated in Fig 6.

### Machine learning algorithms

#### Gradient boosting decision tree (GBDT).

GBDT is a powerful ensemble machine learning algorithm. It optimizes the model continuously using a gradient descent method to minimize the prediction error of the current model. GBDT computes the residuals between the predicted and actual values of the current model in each iteration. It trains a decision tree to fit the residuals. The predictions generated by the decision tree are combined with the predictions of all prior trees to create a new model. This process is repeated to improve the predictive accuracy of the model [36]. The model’s prediction at iteration *t* is given by:


$$F_{t}(x)=F_{t-1}(x)+\eta h_{t}(x)$$ (3)

where *F*_*t*_*(x)* is the model’s prediction, *h*_*t*_*(x)* denotes the newly added tree, and *η* is the learning rate.

#### Gaussian Naive Bayes (GNB) algorithm.

A Bayesian classifier is a fundamental classification algorithm in machine learning. The assumption is that the conditional probabilities of the feature dimensions of a sample follow a Gaussian distribution. It computes the posterior probability that a new sample belongs to a given category based on its feature distribution. The category with the highest posterior probability is selected [37]. The GNB classifier is particularly effective with continuous variables and assumes that the samples have a Gaussian or normal distribution. The probability density function of the normal distribution is utilized to calculate the probabilities.


$$P(x_{i}/y)=\frac{1}{\sqrt{2\pi y\sigma_{y}^{2}}}exp(-\frac{x_{i}-\mu_{y}^{2}}{2\sigma_{y}^{2}})$$ (4)

where *µ*_*y*_ denotes the mean of feature *x*_*i*_ in a sample of category *y*; and *σ*_*y*_ denotes the standard deviation of feature *x*_*i*_ in a sample of category *y*.

#### Logistic regression (LR).

LR is a generalized linear model for binary classification [38]. It handles binary dependent variables using logistic functions and employs a cost function to optimize model parameters iteratively. The model’s quality is assessed by testing. This method has several advantages, including high processing speed, simplicity, ease of interpretation, and feature weight assignment. It can be updated to incorporate new data. However, it has limitations regarding adaptability to diverse datasets and scenarios, making it less versatile than decision tree algorithms.

#### Multilayer perceptron (MLPC).

The MLPC, also known as an ANN, is an improvement of the single-layer perceptron and has multiple layers of neurons [39]. Typically, an MLPC consists of three layers. The first layer is the input layer, the middle layer is the hidden layer, and the last layer is the output layer. Importantly, there is no limit to the number of hidden layers, allowing flexibility for different processing requirements. Similarly, there are no restrictions on the number of neurons in the hidden layers or the output layer. The neurons in the hidden layers receive input from the preceding hidden layers. They process the information and transmit the output to the neurons in the subsequent hidden layers [40].

#### Random forest (RF).

RF is a classification algorithm proposed by Leo Breiman [41]. It uses boot-strapping or sampling with replacement, which is performed repeatedly on the training dataset ‘N’ to generate a new set of training samples. These new samples are used to train individual decision trees. This process is repeated, and ‘m’ decision trees are created to form an RF. The algorithm determines how many decision trees classify the data into categories, and the final classification result is determined by the majority vote among the classification trees. We used RF to evaluate landslide susceptibility due to its ability to handle complex datasets and generate stable predictions.

The modeling process has the following steps:

(1) Select *n* samples from the sample set using sampling with replacement;
(2) Randomly select k features and create a decision tree using the selected samples (usually CART is used, but another algorithm can be used);
(3) Repeat the above two steps m times to generate m decision trees, forming an RF;
(4) The final classification result is determined by voting.

#### Support vector machine (SVM).

SVM refers to a class of generalized linear classifiers used for binary classification in supervised learning. Its distinguishing feature is the maximum margin hyperplane, the decision boundary based on the training samples. An SVM transforms the classification problem into solving convex quadratic functions. The hyperplane is a decision boundary in the feature space. It is defined as $w^{T}x+b=0\;$where w is the weight vector, x represents the feature vector, and b is the bias term. SVM optimizes separation by minimizing the norm of the weight vector ${\left\|w\right\|}^{2}$, which is equivalent to maximizing the margin. There are constraints to ensure the correct classification of training samples. In cases where the data is linearly separable, the SVM identifies the optimal classification hyperplane in the feature space. However, when the data are linearly inseparable, SVM uses relaxation variables and nonlinear mapping to project the samples from a low-dimensional to a higher-dimensional feature space. This transformation enables the linear separation of the samples in the higher-dimensional space, enabling the identification of the optimal classification hyperplane.

The slope disaster conditions were the dependent variable, and the nine influencing factors were independent variables. Slope units were employed for assessing the distribution and performance of landslide susceptibility [42]. The SMOTE-Tomek sampling technique was employed to create a balanced sample set. This set was divided into a training set (70%) and a test set (30%). The models were trained using training data, and the predictive performance was evaluated using several metrics. A grid search was implemented with k-fold cross-validation to optimize the hyperparameter settings. This approach enabled the definition of different hyperparameter values or ranges for different models, facilitating performance assessment for different data types. These strategies mitigated overfitting and improved the models’ generalizability.

The optimum model was selected for further analysis. The susceptibility index was calculated based on the machine learning results and classified using natural breaks for visualization in ArcMap. In this study, we employed a combination of grid search and K-fold cross-validation for hyperparameter tuning. First, we defined the search space for the hyperparameters of each machine learning model and used grid search to test various combinations of these parameters. Then, we validated each combination using 5-fold cross-validation to ensure the model’s stability and generalizability. By comparing the performance of different hyperparameter configurations, we selected the optimal settings for each model. Table 4 provides a summary of the key hyperparameters and their values or ranges for different models.

**Table 4:** Machine learning model hyperparameters and their values.

Model	Hyperparameter	Value or Range
**GBDT**	Number of Trees	100
	Learning Rate	0.1
	Maximum Depth	5
	Subsample	0.8
**GNB**	Assumed Distribution	Gaussian
**LR**	Regularization Parameter	1
	Maximum Iterations	100
**MLPC**	Number of Hidden Layers	100
	Activation Function	ReLU
	Learning Rate	Adaptive
	Maximum Iterations	200
**RF**	Number of Trees	100
	Maximum Depth	None
	Maximum Features	Sqrt
	Minimum Samples for Splitting	2
**SVM**	Kernel Function	RBF
	Regularization Parameter	1
	Kernel Coefficient	Scale

### Model performance indicators

The model performance was assessed using accuracy, F1 score, ROC, and the area under the ROC curve (AUC). These metrics provide insights into the classifiers’ efficacy in distinguishing between landslide and non-landslide areas.

Accuracy is the percentage of correctly predicted results relative to the total number of samples, making it a reliable indicator of model performance, particularly when the samples are balanced. Accuracy is expressed as:


$$Accuracy=\frac{TP+TN}{TP+FP+FN+TN}$$ (5)

where *TP* denotes true positives, *TN* represents true negatives, *FP* indicates false positives, and *FN* refers to false negatives. Accuracy provides a straightforward measure of the proportion of correctly classified instances, reflecting the overall effectiveness of the model.

The F1 score is defined as the harmonic mean of precision and recall, providing a unified metric that balances these two aspects of model performance, especially in the presence of high FN rates. It is calculated as:


$$F1=2*\frac{Recall*Precision}{Recall+Precision}$$ (6)

$$Precision=\frac{TP}{TP+FP},Recall=\frac{TP}{TP+FN}$$ (7)

The ROC curve is a widely utilized metric for evaluating model performance. It plots sensitivity (true positive rate (TPR)) on the Y-axis and specificity (true negative rate (TNR)) on the X-axis. A curve that approaches the upper-left corner indicates superior classification performance. The area under the ROC curve (AUC) is typically employed as an overall performance criterion; a larger AUC value corresponds to better model performance and vice versa.

In addition to evaluating the model’s predictive performance, an important task in susceptibility assessment is to assess the reliability of the landslide area. The susceptibility intensity index was used to assess the reliability of the susceptibility classes to determine the optimal susceptibility prediction model. It is expressed as follows:


$$R_{i}=\frac{L(P_{i})}{A(P_{i})}$$ (8)

where *R*_*i*_ represents the landslide susceptibility index of susceptibility class *i*, *L(pi)* represents the ratio of the number of historical landslides located in the zone of susceptibility class *i* to the number of total historical landslides, and *A(Pi)* represents the ratio of the area of susceptibility class *i* to the total study area.

### Interpretive analytical methods: SHAP

The SHAP model, inspired by Shapley values in game theory, was developed by Lundberg and Lee [43]. It computes the marginal contribution of each feature to the output of a machine learning model. It aims to explain the predictions of ‘black-box models’ globally and locally. SHAP is a powerful tool for interpreting machine learning models, particularly complex ones, and is crucial in assessing the contributions of influencing factors [44]. It provides insights into the factors’ significance levels and rankings in influencing landslide susceptibility evaluations. SHAP visualization enhances our understanding of the factors’ contributions to the predicted outcomes, clarifying the model results. During the training or testing phases of a machine learning model, the SHAP model generates predictions for instances and assigns value to features, representing their contributions to the prediction result.


$$\emptyset_{m}=\sum_{L⊑N\left\{m\right\}}\frac{|L|!(M-|L|-1)!}{M!}\times \left[\nu (L\cup \left\{m\right\})-\nu (L)\right]$$ (9)

where *ϕ*_*m*_ is the contribution of the m-th feature, *L* is the subset of features, N{m} is the set of features, *M* is the total number of input features, ν(L∪{m}) is the predicted value of the model when the samples have feature values in L∪{m}, and ν(L) is the predicted value of the model when the samples have only eigenvalues *L*. Based on the additive eigenvalue approach, a linear function is obtained:


$$g(x)=\phi_{0}+\sum_{m=1}^{M}\phi_{m}x_{m}$$ (10)

where *g(x)* is the post hoc explanatory model prediction for sample *x*, *ϕ*_*0*_ is the mean of the model predictions, and *x*_*m*_ is the m-th feature sample.

## Results and discussion

### Comparison of the six machine learning models

Table 5 lists the evaluation metrics, including accuracy and F1 score, for the models. All models have relatively high classification performance. The GBDT model achieves the highest performance, with an accuracy of 0.823 and an F1 score of 0.740, reflecting a well-balanced capability in classifying positive and negative samples. The RF model delivers comparable results, with an accuracy of 0.791 and an F1 score of 0.723, highlighting its robust performance and suitability for demanding classification tasks. In contrast, the SVM, MLPC, and GNB models exhibit moderate performance. The LR has the lowest accuracy and F1 values, indicating it low effectiveness for this classification task.

**Table 5:** Comparison of model evaluation indicators.

Model	Indicators		Model	Indicators	
**GBDT**	Accuracy	0.823	**MLPC**	Accuracy	0.683
	F1	0.740		F1	0.615
**GNB**	Accuracy	0.722	**RF**	Accuracy	0.791
	F1	0.715		F1	0.723
**LR**	Accuracy	0.523	**SVM**	Accuracy	0.701
	F1	0.545		F1	0.719

**Fig 7:**
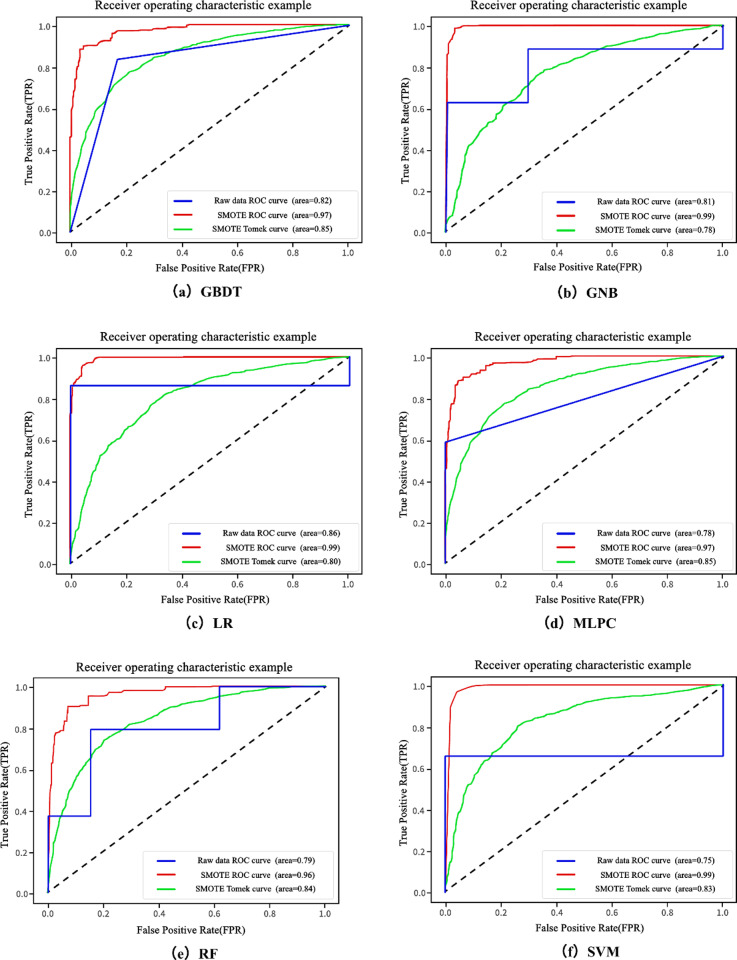
ROC evaluation results.

Fig 7 shows the ROC results. The ROC curves generated from the raw data and the SMOTE and SMOTE-Tomek sampling indicate that all machine learning models trained on the enhanced datasets accurately predicted landslide susceptibility areas. The GBDT model exhibited the highest predictive accuracy for landslide sensitivity, with AUC values of 0.82 for raw data, 0.97 for SMOTE, and 0.86 for SMOTE-Tomek. These results suggest a high stability and generalizability of the models. Although the AUC values for the six models based on the raw data exceeded 0.70, the ROC curves were less smooth with a stepped pattern and were closer to the diagonal line than those derived from the other two sampling methods. This finding indicates that these models exhibited lower sensitivity and specificity than the SMOTE and SMOTE-Tomek approaches [45]. A comparative analysis revealed that SMOTE-Tomek consistently outperformed SMOTE for all six models, highlighting its efficacy in enhancing model performance for landslide susceptibility prediction.

### Accuracy of landslide susceptibility classes

**Fig 8:**
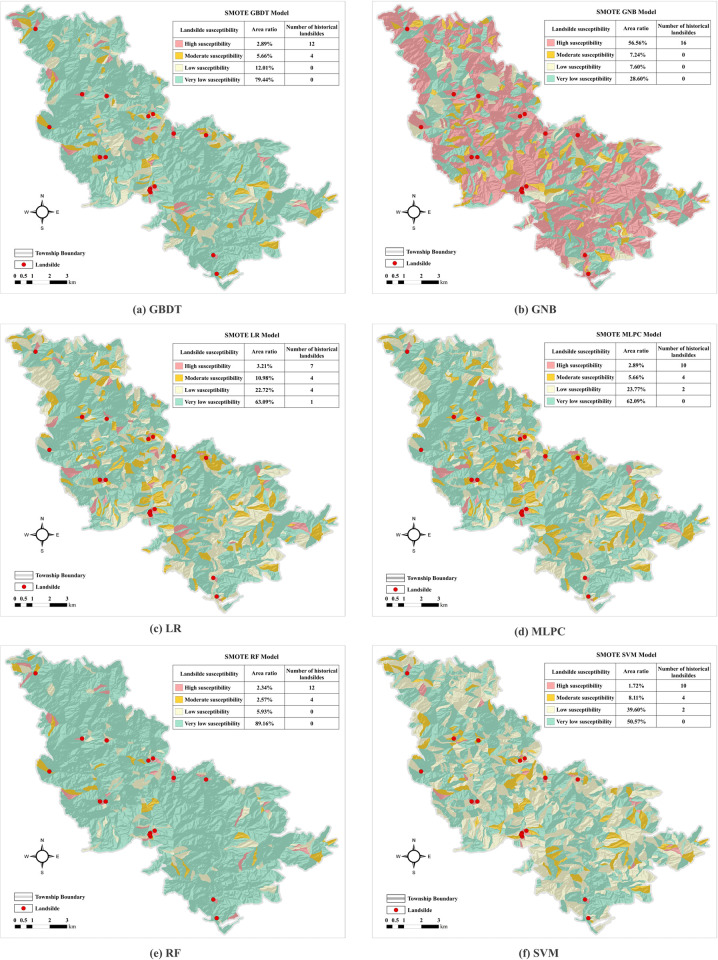
Landslide susceptibility maps derived from SMOTE sampling. (Republished from the data provided by Zhejiang Zhenan Comprehensive Engineering Surveying Institute under a CC BY license).

**Fig 9:**
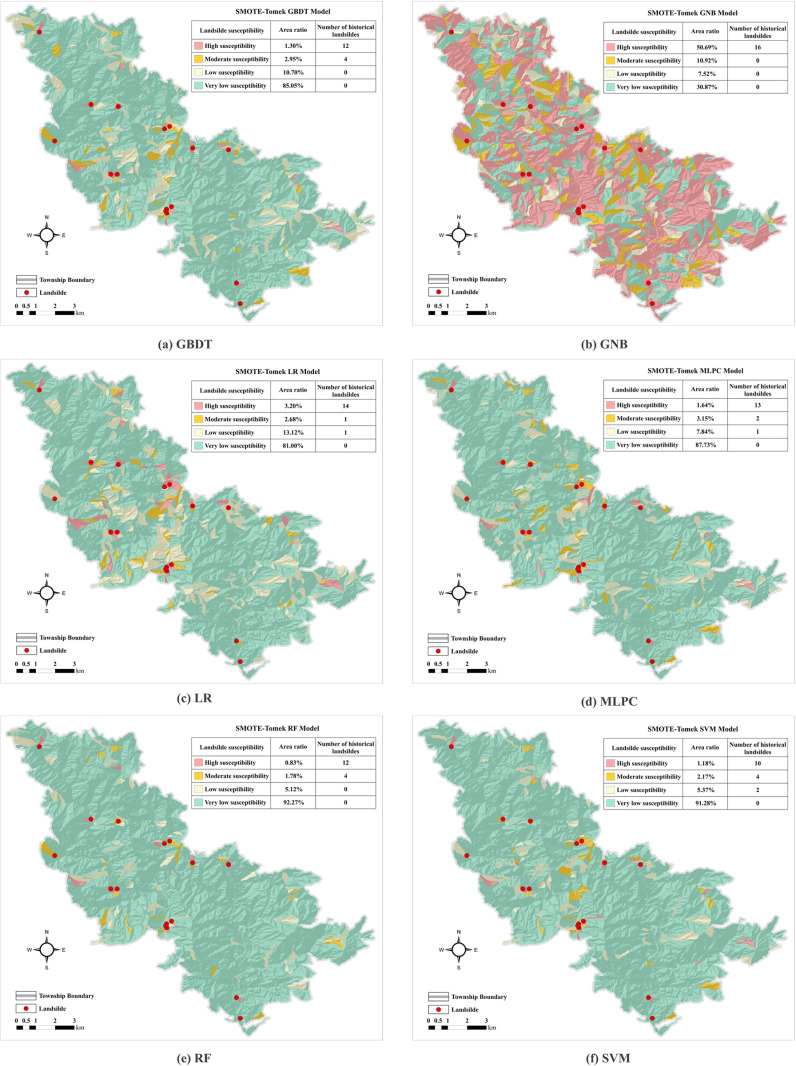
Landslide susceptibility maps derived from SMOTE Tomek-sampling. (Republished from the data provided by Zhejiang Zhenan Comprehensive Engineering Surveying Institute under a CC BY license).

The landslide susceptibility maps under different sampling conditions are presented in Figs 8 and 9. The results indicate that the majority of Liandu District is categorized as having very low and low landslide susceptibility, with only a few high susceptibility zones. Most historical landslides are concentrated in these high-susceptibility areas, and there are very few incidents in other susceptibility categories. Compared to previous models that used only machine learning methods, this outcome demonstrates the effectiveness of the SMOTE-Tomek sampling method combined with six machine learning models in producing reliable and accurate assessments of landslide susceptibility in this region.

The area ratio and historical landslide numbers in different susceptibility categories for six machine learning models using raw data and the datasets after SMOTE and SMOTE-Tomek sampling are summarized in Tables 6–8. The area ratio of the GNB model increased from very low to high susceptibility. Conversely, the values of the other five models decreased from very high to low susceptibility, indicating that the GNB model may overestimate landslide susceptibility. Moreover, most of the predicted landslides were located in moderate and high susceptibility areas, demonstrating the reliability and validity of the model predictions. The SMOTE-Tomek method combined with the five models resulted in a significantly larger area of very low susceptibility, whereas the area ratio of high susceptibility was the smallest. Most landslide points were located in the high susceptibility zone, in agreement with the actual landslide distribution in Liandu District. This consistency suggests that SMOTE-Tomek sampling provides a feasible approach for assessing landslide susceptibility.

**Table 6:** Area ratio and historical landslide numbers in different susceptibility categories for different models using raw data.

Model	Area ratios in different susceptibility categories (%)				Landslide numbers in different susceptibility categories			
	Very low	Low	Medium	High	Very low	Low	Medium	High
**GBDT**	68.59%	1.06%	15.21%	15.14%	3	0	1	12
**GNB**	29.52%	8.73%	15.72%	46.03%	0	0	0	16
**LR**	63.68%	25.01%	8.51%	2.80%	0	2	5	9
**MLPC**	52.81%	26.58%	12.47%	8.14%	1	0	3	12
**RF**	43.14%	24.71%	24.61%	7.54%	0	0	0	16
**SVM**	52.81%	26.58%	12.47%	8.14%	0	0	5	11

**Table 7:** Area ratio and historical landslide numbers in different susceptibility categories for different models using the SMOTE sampling method.

Model	Area ratio in different susceptibility categories (%)				Landslide numbers in different susceptibility categories			
	Very low	Low	Medium	High	Very low	Low	Medium	High
**GBDT**	79.44%	12.01%	5.66%	2.89%	0	0	4	12
**GNB**	28.60%	7.60%	7.24%	56.56%	0	0	0	16
**LR**	63.09%	22.72%	10.98%	3.21%	1	4	4	7
**MLPC**	62.09%	23.77%	11.56%	2.58%	1	2	4	10
**RF**	89.16%	5.93%	2.57%	2.34%	0	0	4	12
**SVM**	50.57%	39.60%	8.11%	1.72%	0	2	4	10

**Table 8:** Area ratio and historical landslide numbers in differentsusceptibility categories for different models using the SMOTE-Tomek sampling method.

Model	Area ratio in different susceptibility categories (%)				Landslide numbers in different susceptibility categories			
**Very low**	**Low**	**Medium**	**High**	**Very low**	**Low**	**Medium**	**High**
**GBDT**	85.05%	10.70%	2.95%	1.30%	0	0	4	12
**GNB**	30.87%	7.52%	10.92%	50.69%	0	0	0	16
**LR**	81.00%	13.12%	2.68%	3.20%	0	1	1	14
**MLPC**	87.73%	7.48%	3.15%	1.64%	0	1	2	13
**RF**	92.27%	5.12%	1.78%	0.83%	0	0	4	12
**SVM**	91.28%	5.37%	2.17%	1.18%	0	2	4	10

**Fig 10:**
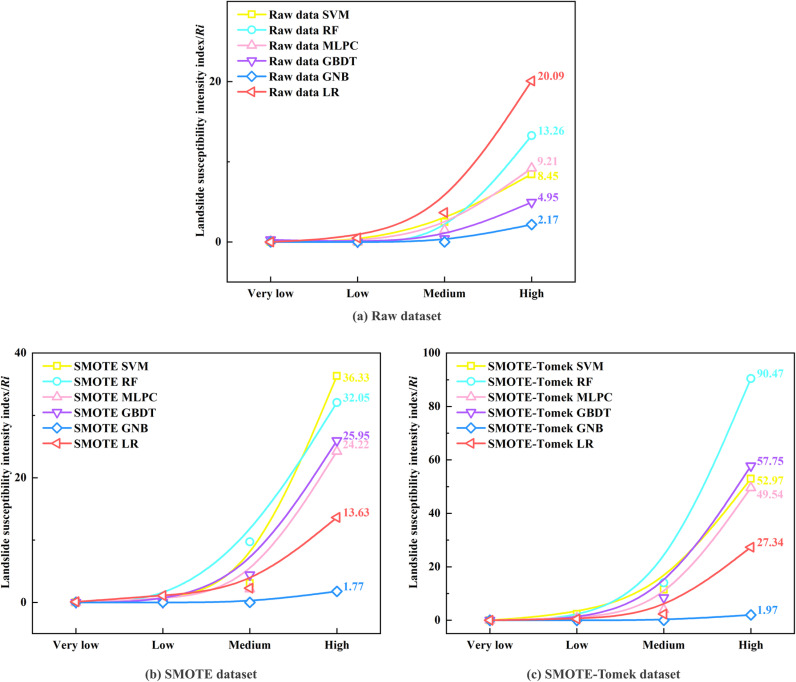
Susceptibility intensity index.

The susceptibility intensity index is presented in Fig 10. Regardless of whether the raw data, SMOTE sampling, or SMOTE-Tomek sampling were used, the *R*_*i*_ for the SVM, RF, MLPC, GBDT, and LR models increased from very low to high susceptibility. This trend indicates the effectiveness of combining the three sampling methods with the five models for landslide susceptibility prediction in the study area. For the raw dataset, the LR model exhibited the highest Ri value in the high susceptibility zones, followed by the MLPC and RF models. For the SMOTE oversampled dataset, the SVM and RF models had higher *R*_*i*_ values in the high susceptibility areas, suggesting these models provide higher accuracy. In contrast, for the dataset enhanced by the SMOTE-Tomek method, the RF model yielded the highest *R*_*i*_ in the high-susceptibility zones, indicating its superior stability and robustness for different sampling methods. In general, a higher susceptibility intensity index in the high-susceptibility zones indicates that the classification is in better agreement with actual conditions. Thus, the RF is highly suitable for large-scale landslide susceptibility assessments. Although the accuracy and F1 score of RF are slightly lower than those of GBDT, the reliability of RF in susceptibility prediction justifies its use for subsequent factor analyses. Therefore, the subsequent analysis was conducted using the RF model results.

### Interpretability of RF model using SHAP

**Fig 11:**
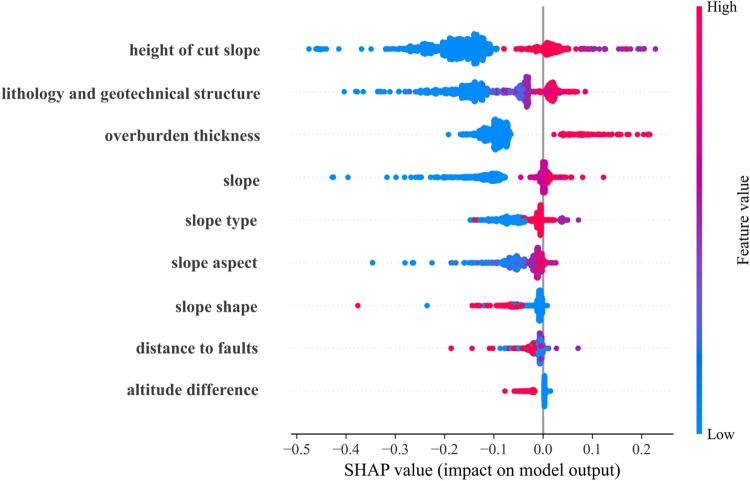
Factor importance of the SMOTE-Tomek-RF model.

**Fig 12:**
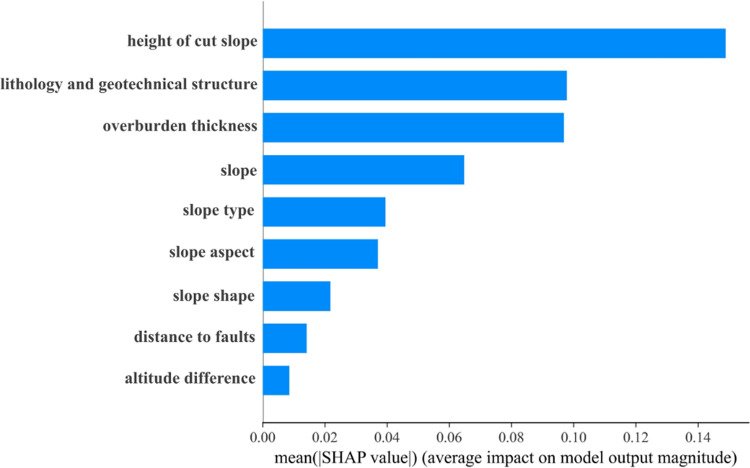
Importance of evaluation factors of the RF model.

Geological and topographic features affect landslide susceptibility. Figs 11 and 12 present the global interpretation of SHAP values for factors influencing landslide susceptibility prediction using the RF model. The height of cut slopes was the most significant factor. Regions with higher cut slopes exhibited a markedly increased landslide risk due to greater gravitational forces and potential sliding forces, significantly reducing slope stability. The second most critical factor was lithology and geotechnical structure. Areas with soft rock formations and near fault zones exhibited higher landslide susceptibility. This result is primarily attributed to the lower shear strength of soft rock and the fractured rock masses caused by faults, which compromised geological stability. Additionally, the overburden thickness substantially affected landslide susceptibility. Rainfall or groundwater can destabilize thick overburden layers, particularly when soil cohesive strength is relatively low, increasing the likelihood of landslides. In contrast, the effects of slope type and slope aspect are more complex. Slope type affects the patterns of rock mass failure and instability, whereas the slope aspect influences spatial variability in landslide risk due to differences in rainfall distribution and weathering.

The height of the cut slope, lithology and geotechnical structure, overburden thickness, and slope were the primary factors influencing landslide susceptibility. Most SHAP values were positive, indicating a substantial positive contribution to the model’s predictive performance.

**Fig 13:**
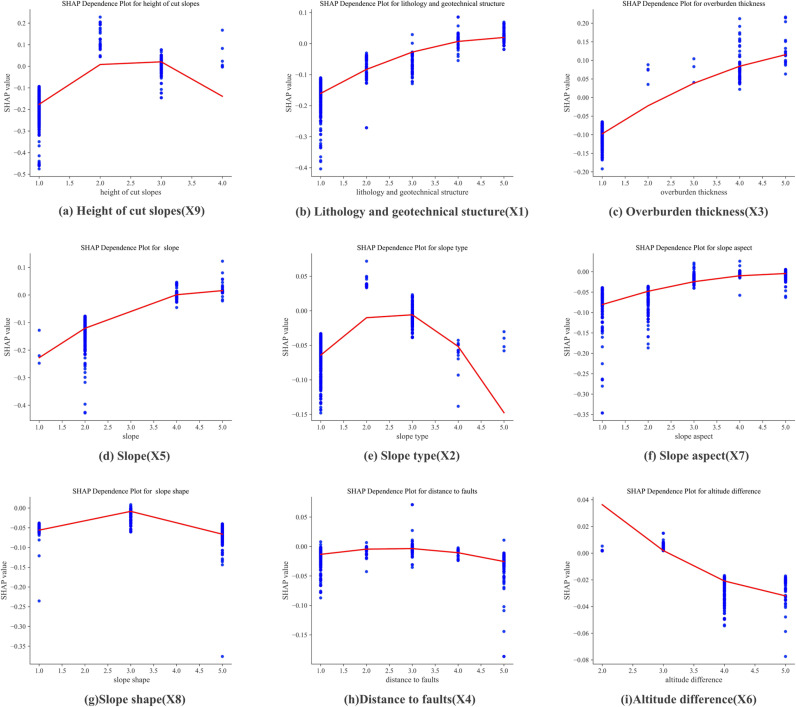
Effects of individual factors on landslide susceptibility.

We investigated the relationships between the SHAP values and the features with significant impacts on landslide, focusing on those that are relatively easy to obtain in geological surveys, the height of the cut slope, lithology and geotechnical structure, overburden thickness, and slope. The impacts of the factors and their SHAP values are presented in Fig 13. The SHAP values for the height of the cut slope exhibit a nonlinear relationship. As the height increased from 1.0 to 2.5, the susceptibility rose markedly, with SHAP values peaking around 0.2. Beyond this threshold, the SHAP values declined at higher slope heights, indicating a potential threshold effect where susceptibility decreased beyond a certain point. In contrast, the SHAP values for lithology and geotechnical structure and the overburden thickness showed a consistent upward trend, indicating a strong positive correlation between these features and landslide susceptibility. Higher lithological complexity or weaker geotechnical properties significantly increased the risk. Similarly, thicker overburden layers were related to higher landslide susceptibility, highlighting their destabilizing effect on slopes. The SHAP values increased from −0.2 to 0.1 as the slope gradient rose from 1.0 to 5.0. This finding underscores the substantial effect of slope steepness on landslide susceptibility, with higher slopes associated with higher risk.

Our machine learning models, informed by this dataset, predicted landslide susceptibility across the area, with the results showing that 92.27% of the region falls into the very low susceptibility zone, and 5.12% into the low susceptibility zone. Notably, the high sensitivity zone, where most historical landslides have occurred, accounts for only 0.83% of the area. The model highlights that cut slope height is the most significant factor influencing landslide sensitivity, which corresponds well with the concentration of historical landslides in areas with similar geological conditions. Therefore, effective monitoring and precise management should be implemented in areas near high cut slopes, soft rock formations, thick overburden layers, and fault zones. Development and construction activities in highly susceptible regions should be restricted. Additionally, drainage systems and slope reinforcement measures need to be optimized. Reinforcement techniques, such as anti-slide piles and anchoring, should be tailored to the specific geological conditions to enhance slope stability.

## Conclusions

The analysis of high-resolution landslide susceptibility is required as more refined management policies are implemented. Thus, it is necessary to propose a framework to address data imbalance and achieve high prediction accuracy. We compared six commonly used machine learning algorithms using geological survey data from Taiping Township. The SMOTE-Tomek hybrid sampling method was employed to address class imbalance. The main conclusions are as follows:

1. The SMOTE-Tomek hybrid sampling method effectively mitigated data imbalance, significantly improving model performance. This approach enhanced classification accuracy across all tested algorithms and provided a robust solution for handling imbalanced datasets in landslide susceptibility analysis.
2. The comparative evaluation of six machine learning algorithms demonstrated strong predictive capabilities in landslide susceptibility classification. Among them, the Gradient Boosting Decision Tree (GBDT) and Random Forest (RF) models exhibited the highest accuracy and F1 scores, outperforming traditional susceptibility assessments that often rely on expert judgment and subjective delineations.
3. The RF model achieved superior predictive performance, with susceptibility classifications aligning well with actual conditions. The proportion of land classified as high, medium, low, and very low susceptibility accounted for 92.27%, 5.12%, 1.78%, and 0.83% of the total study area, respectively, highlighting the model’s ability to differentiate risk levels with high precision.
4. SHAP analysis was employed to quantify the contribution and nonlinear effects of key influencing factors. The most significant determinants of landslide susceptibility were the height of the cut slope, lithology and geotechnical structure, overburden thickness, and slope gradient. The identified factor importance was consistent with expert-derived weight assessments, reinforcing the model’s interpretability and reliability.

This study conducted a landslide susceptibility assessment at the township level by integrating data balancing techniques with machine learning methods. We analyzed 1,325 landslide samples from Taiping Township, Zhejiang Province, using SMOTE and Tomek linking to balance the dataset. Our results demonstrate the high predictive accuracy of machine learning algorithms, particularly the Random Forest model. These findings underscore the need for targeted risk management strategies and offer practical insights for effective disaster risk reduction and mitigation in similar geological settings.

This study has several limitations that should be addressed in future research. We utilized data from a single township. Future studies should conduct horizontal comparisons of different regions and consider a broader range of geographic characteristics. Additionally, there remains potential for algorithmic advancements. Research has indicated that more sophisticated algorithms exhibit superior performance. Future studies should validate various algorithms for landslide susceptibility and hazard assessments.
